# Supply-side and demand-side policies for biosimilars: an overview in 10 European member states

**DOI:** 10.1080/20016689.2017.1307315

**Published:** 2017-04-28

**Authors:** Cécile Rémuzat, Anna Kapuśniak, Aleksandra Caban, Dan Ionescu, Guerric Radière, Cyril Mendoza, Mondher Toumi

**Affiliations:** ^a^ Pricing and Market Access Department, Creativ-Ceutical, Paris, France; ^b^ Pricing and Market Access Department, Creativ-Ceutical, Krakow, Poland; ^c^ Global Pricing and Market Access Biopharmaceuticals Department, Sandoz International GmbH, Holzkirchen, Germany; ^d^ Laboratoire de Santé Publique, Aix-Marseille Université, Université de la Méditerranée, Marseille, France

**Keywords:** Biosimilar, policies, pricing, savings, uptake

## Abstract

Objective: This study aimed to provide an overview of biosimilar policies in 10 EU MSs.

**Methods**: Ten EU MS pharmaceutical markets (Belgium, France, Germany, Greece, Hungary, Italy, Poland, Spain, Sweden, and the UK) were selected. A comprehensive literature review was performed to identify supply-side and demand-side policies in place in the selected countries.

**Results**: Supply-side policies for biosimilars commonly include price linkage, price re-evaluation, and tendering; the use of internal or external reference pricing varies between countries; health technology assessment is conducted in six countries. Regarding demand-side policies, pharmaceutical prescription budgets or quotas and monitoring of prescriptions (with potential financial incentives or penalties) are in place in eight and in seven countries respectively. Switching is generally allowed, but is solely the physician’s responsibility. Automatic substitution is not recommended, or even forbidden, in most EU MSs. Prescription conditions or guidelines that apply to biosimilars are established in nearly all surveyed EU MSs.

**Conclusions**: Important heterogeneity in policies on biosimilars was seen between (and even within) selected countries, which may partly explain variations in biosimilar uptake. Supply-side policies targeting price have been reported to limit biosimilar penetration in the long term, despite short-term savings, while demand-side policies are considered to positively impact uptake.

## Background and objective

The global economic crisis, which has affected Europe since mid-2007, forced the European Union (EU) member states (MSs) to implement several cost-containment measures to decrease or control healthcare expenditure, with major pressure exerted on pharmaceutical budgets [1,2]. The introduction of new innovative and expensive medicines, increasing demand for better healthcare, the growing prevalence of chronic diseases, and population ageing are expected to add up, placing a substantial burden on health insurance systems [3–5].

Increasing the use of generics and biosimilars is considered an efficient way to improve patient health outcomes at a reduced cost, while sparing the budget for innovative medicines [6–10]. However, while the generic market has gained maturity, the biosimilar market is still immature, with just 10 years having elapsed since the first biosimilar approval in the EU (2006) [11]. With the growing budget impact of costly biologic medicines – constituting about 27% of pharmaceutical sales in Europe [12] in 2014 – biosimilar promises for healthcare cost savings are thoroughly scrutinized by healthcare payers. In 2016, IMS Health estimated the cumulative potential savings in the top five EU markets (France, Germany, Italy, Spain, and the UK) and the USA at around 50–100 billion EUR over the next 5 years [6]. The extent of cost savings will depend on the implementation of policies supporting biosimilar uptake in each individual country. At present, the EU has the most developed regulated biosimilars market, with 21 medicines already authorized as of June 2016 [13] (Table 1).

**Table 1.** List of biosimilars approved by the European Medicines Agency (as of June 2016) [13].

Medicine class	Reference medicine brand name (company)	Biosimilar brand name	International non-proprietary name	Marketing authorization holder	Marketing authorization date
Granulocyte-colony stimulating factor (G-CSF)	Neupogen® (Amgen)	Accofil®	Filgrastim	Accord Healthcare Ltd	18 September 2014
	Neupogen® (Amgen)	Grastofil®	Filgrastim	Apotex Europe BV	18 October 2013
	Neupogen® (Amgen)	Nivestim®	Filgrastim	Hospira UK Ltd	8 June 2010
	Neupogen® (Amgen)	Zarzio®	Filgrastim	Sandoz GmbH	6 February 2009
	Neupogen® (Amgen)	Filgrastim Hexal®	Filgrastim	Hexal AG	6 February 2009
	Neupogen® (Amgen)	Biograstim®	Filgrastim	AbZ-Pharma GmbH	15 September 2008
	Neupogen® (Amgen)	Ratiograstim®	Filgrastim	Ratiopharm GmbH	15 September 2008
	Neupogen® (Amgen)	Tevagrastim®	Filgrastim	Teva GmbH	15 September 2008
Epoetin	Eprex/Erypo® (Janssen)	Retacrit®	Epoetin zeta	Hospira UK Ltd	18 December 2007
	Eprex/Erypo® (Janssen)	Silapo®	Epoetin zeta	Stada Arzneimittel AG	18 December 2007
	Eprex/Erypo® (Janssen)	Abseamed®	Epoetin alfa	Medice Arzneimittel Pütter GmbH & Co. KG	28 August 2007
	Eprex/Erypo® (Janssen)	Epoetin alfa Hexal®	Epoetin alfa	Hexal AG	28 August 2007
	Eprex/Erypo® (Janssen)	Binocrit®	Epoetin alfa	Sandoz GmbH	28 August 2007
Insulin	Lantus® (Sanofi)	Abasaglar®	Insulin glargine	Eli Lilly Regional Operations GmbH	9 September 2014
Anti-tumour necrosis factor (anti-TNF)	Enbrel® (Pfizer)*	Benepali®	Etanercept	Samsung Bioepis UK Ltd (SBUK)	14 January 2016
	Remicade® (Janssen)	Flixabi®	Infliximab	Samsung Bioepis UK Ltd (SBUK)	26 May 2016
	Remicade® (Janssen)	Inflectra®	Infliximab	Hospira UK Ltd	10 September 2013
	Remicade® (Janssen)	Remsima®	Infliximab	Celltrion Healthcare Hungary Kft.	10 September 2013
Gonadotropins	Gonal-f® (Merck Serono)	Bemfola®	Follitropin alfa	Finox Biotech AG	27 March 2014
	Gonal-f® (Merck Serono)	Ovaleap®	Follitropin alfa	Teva Pharma BV	27 September 2013
Human growth hormone (hGH)	Genotropin® (Pfizer)	Omnitrope®	Somatropin	Sandoz GmbH	12 April 2006

Policy makers in the EU MSs have implemented and are discussing policies to regulate the pricing and reimbursement of biosimilars and to enhance biosimilar uptake. These policies can be divided into supply-side and demand-side policies. Supply-side policies are measures that are primarily directed at specific healthcare system stakeholders who are responsible, for example, for the pricing and reimbursement of medicines. These include policies such as price regulation, health technology assessment (HTA), and procurement conditions. Demand-side policies are measures that are directed towards stakeholders who prescribe, dispense, and ask for medicines, and include incentives for physicians, pharmacists, and patients [14,15].

A wide difference in biosimilar uptake has been reported across Europe and among the six therapeutic biosimilar classes, i.e. granulocyte–colony-stimulating factor (G-CSF), epoetin (EPO), insulin, anti-tumour necrosis factor (anti-TNF), gonadotropins, and human growth hormone (hGH) [16]. The biosimilar market is rapidly evolving and attractive, warranting an investigation of applicable policies. The objective of this study was to provide an updated overview of supply-side and demand-side policies on biosimilars in the 10 EU MSs with the highest pharmaceutical expenditure (Belgium, France, Germany, Greece, Hungary, Italy, Poland, Spain, Sweden, and the UK), and to discuss the potential impact of these policies on the biosimilar market and its future perspectives.

## Methods

Ten EU MS pharmaceutical markets were studied (Belgium, France, Germany, Greece, Hungary, Italy, Poland, Spain, Sweden, and the UK), based on the country selection performed for a quantitative study that we previously conducted to assess key drivers for biosimilar uptake [17]. The countries with the highest pharmaceutical expenditure were selected for the analysis. A comprehensive literature review was performed to identify publications describing the supply-side and demand-side policies in place in the selected EU MSs.

The search was performed in the following databases: MEDLINE, Embase, The Cochrane Library, Generics and Biosimilars initiative (GaBi) journal and website, and, for conference abstracts, the International Society for Pharmacoeconomics and Outcomes Research (ISPOR) and Health Technology Assessment International (HTAi) websites. The search strategy used the free search terms presented in Table 2. Our review was supplemented by additional searches in national health authorities and parliamentary websites, Google and Google Scholar, and proprietary databases.

**Table 2.** Terms used to search particular databases and publications dated between 2005 and September 2016.

Database	Search terms
MEDLINE	Biosimilar, off-patent biologic, follow-on biologic, similar biologic, similar biotechnological, subsequent entry biologic, incentive, uptake, penetration, policy, pricing, reimbursement, tender, reference pricing, price referencing, price linkage, health technology assessment, INN prescribing, purchase, procurement, demand-side policy, supply-side policy, substitution, Europe, European Union, Germany, France, Italy, Spain, Sweden, Poland, Hungary, Belgium, Greece, United Kingdom, UK
Embase	
Cochrane Library	
GaBi Journal and GaBi website	Biosimilar, incentive
ISPOR	Biosimilar
HTAi	Biosimilar, off-patent biologic, follow-on biologic, subsequent entry biologic, incentive

The search was conducted in the English language for all databases, except in country-specific databases (national health authorities and parliamentary websites), which were searched using the local language. Publications were searched from 2005 [the year of establishment of the regulatory framework for the development of biosimilars by the European Medicines Agency (EMA) [18]] to mid-September 2016.

Initially, articles published between 2005 and November 2015 were screened. Thereafter, monitoring of any new publications to be included in the scope of this search was conducted in each database on a monthly basis until September 2016.

In the second stage of the review, publications were screened and selected for relevance to the topic and the full-text papers were examined in detail to identify supply-side and demand-side policies in each country. Policies were classified as supply or demand side as follows:

Supply-side policies: (1) internal reference pricing (IRP);^1^ ^1^Internal reference pricing (IRP): the practice of using the price(s) of medicines (ATC 5 or ATC 4 level) in a country to set the price or reimbursement of the medicine in a given country [14]. (2) external reference pricing (ERP);^2^ ^2^External reference pricing (ERP): the practice of using the price(s) of a medicine in one or several countries to set the price or reimbursement of the medicine in a given country [14]. (3) HTA; (4) price linkage with the reference medicine (mandatory or not); (5) price re-evaluation; and (6) tendering practices.

Demand-side policies: (1) physician incentives, i.e. pharmaceutical prescription budgets, prescription quotas, monitoring of prescription patterns, financial incentives or penalties, prescription conditions/guidelines, switching,^3^ ^3^Switching: a decision by the treating physician to exchange one medicine for another medicine with the same therapeutic intent in patients who are undergoing treatment [14]. prescribing using international non-proprietary name (INN), education/information; (2) pharmacist incentives, i.e. substitution^4^ ^4^Substitution: the practice of dispensing one medicine instead of another equivalent and interchangeable medicine at the pharmacy level, without consulting the prescriber [91]. right, financial incentives or penalties, education/information; and (3) patient incentives, i.e. patient co-payment, education/information.

## Results

**Figure 1.**
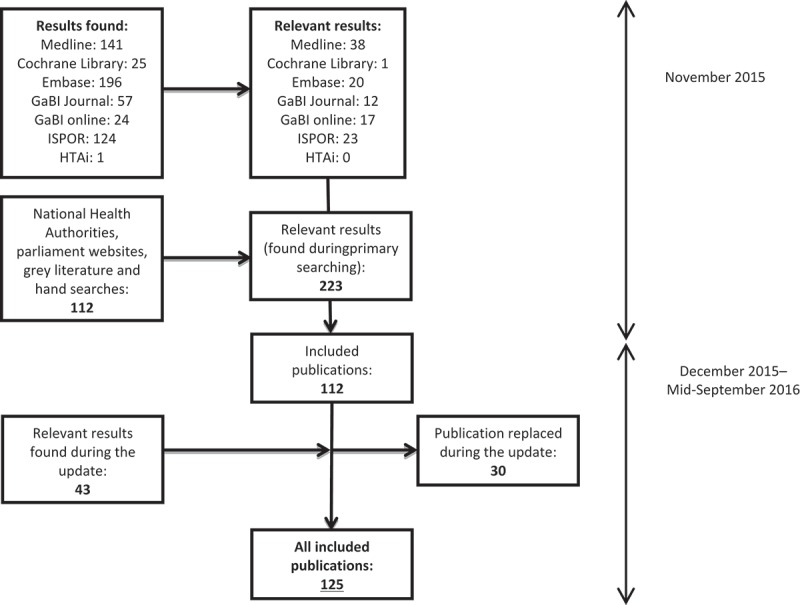
Flowchart of search results.

As of November 2015, the literature search resulted in 680 citations, of which 112 references were considered for inclusion. As of mid-September 2016, 43 new references had been added during the update; 30 of these updated and replaced previous references. Finally, 125 publications were included in this review. The result of the literature review is presented in Figure 1.

### Supply-side policies for biosimilars

An overview of key supply-side policies for biosimilars is presented in Table 3.

**Table 3.** Key supply-side policies for biosimilars.

Policy	Belgium	France	Germany	Greece	Hungary	Italy	Poland^a^	Spain	Sweden	UK
IRP	×	✓	✓	×	✓	×	✓	✓	×	×
ERP	×	×	×	✓	×	✓	✓	×	×	×
HTA	✓	✓	×	×	×	✓	✓^b^	×	✓	✓
Price linkage	✓	✓	✓	×	✓^c^	✓^c^	✓^c^	✓	✓	✓
Price re-evaluation	✓	✓	✓	✓	✓	✓	✓	✓	✓	✓
Tendering	✓	✓	✓	×	✓	✓	✓	✓	✓	✓

IRP: internal reference pricing; ERP: external reference pricing; HTA: health technology assessment; ×: absence of policy; ✓: presence of policy.

^a^ ^ ^Polish regulations do not differentiate between small molecule generics and biosimilars.

^b^ ^ ^Only when there is no equivalent medicine on the market.

^c^ ^ ^Mandatory price reduction (defined price cut).

#### Internal reference pricing [19–24]

IRP is in place for biosimilars in five (France, Germany, Hungary, Poland, and Spain) out of the 10 selected countries, at the fourth Anatomical Therapeutic Chemical (ATC) classification level (therapeutic/pharmacological/chemical subgroup) and/or at the fifth level (chemical substance) [25]. In Hungary, there is also a policy called ‘preferred reference pricing system’, applicable when more than two versions of a biologic medicine are available; this policy sets preferred price ranges based on the reference price for reimbursement, i.e. medicines priced up to 115% of the reference (cheapest) price are eligible for 100% reimbursement [26].

#### External reference pricing [22–24,26,27]

ERP applies to biosimilars in only three of the considered countries (Greece, Italy, and Poland). In Greece, biosimilars are priced at the average of the three lowest prices across the EU, as for new patented medicines. In Italy and Poland, ERP is used as supporting information for pricing reimbursed medicines.

#### Health technology assessment [19,22,23,26]

HTA has been reported for biosimilars in six of the selected countries (Belgium, France, Italy, Poland, Sweden, and the UK). In Belgium, the request for reimbursement for biosimilars follows the same pathway as other pharmaceuticals; these drugs are filed under class 2 medicines (pharmaceuticals with no proven improvement in therapeutic benefit). In France, as for new patented medicines, biosimilars are assessed by the Transparency Committee (similar assessment in actual benefit and no improvement in actual benefit versus the reference medicine) [28]. In Italy, the pricing and reimbursement process takes place at the national level [Italian Medicines Agency (AIFA)], as for other drugs, and HTA may take place at the regional level. In Poland, as for other pharmaceuticals, HTA is employed if there is no reimbursed equivalent medicine on the market [29]. In Sweden, pricing and reimbursement applications have to be sent to the Dental and Pharmaceutical Benefits Agency (TLV); however, the TLV will follow the assessment of the EMA in terms of similarity versus the reference medicine, and will require a cost-minimization model versus the reference medicine. In the UK, the National Institute for Health and Care Excellence (NICE) considers biosimilars in the context of a Multiple Technology Appraisal, in parallel with the corresponding reference medicines used in the same indication. All guidance published for reference medicines in general also applies to relevant biosimilars. If a biosimilar is not included in a technology appraisal, NICE considers preparing a quality-assured summary of the evidence (so called ‘Evidence summary: new medicine’), which critically reviews the evidence but is not a formal guidance [30–32]. The Scottish Medicines Consortium (SMC) does not routinely assess biosimilars on the basis of a full submission if the reference medicine has been accepted by SMC for the same indication in the same population, or was initially licensed and available before 31 January 2002. If the reference medicine was not recommended, full submission for the biosimilar is required [33,34]. Similarly, in Wales, the All Wales Medicines Strategy Group (AWMSG) does not routinely appraise biosimilars if the reference medicine has been accepted by the AWMSG or NICE for the same indication in the same population, or was initially licensed and available before 1 October 2002, and the cost of the biosimilar does not exceed the reference drug’s price. If the reference drug was not appraised, or was not recommended by AWMSG or NICE, the full submission for the biosimilars is needed [35].

#### Price linkage [22–24,26,36]

All selected countries except for Greece, where price setting is based on ERP as described above, link the biosimilar price to the price of the reference medicine, with price reduction being mandatory in three countries (Hungary, Italy, and Poland). In Hungary, the first biosimilar is priced 30% below the reference medicine price, and each subsequent medicine is discounted by another 10%. In Italy, biosimilars are automatically placed in the same reimbursement class as the reference medicine (without any price negotiation with AIFA) only if the proposed price is ‘obvious convenience’. If the manufacturer proposes a higher price, price negotiations are required. The list price of the first biosimilar is discounted between 30% and 75% compared with the price of the reference medicine before patent expiry. The exact discount depends on annual pharmaceutical expenditure on the original pharmaceutical in the past 3 years, and on hospital or retail distribution of the reference medicine [37,38]. In Poland, the price discount for the first biosimilar is 25%.

In the remaining countries, even if price discounts do exist, the rules are more general, and are adjusted on a case-by-case basis with no mandatory price cuts. In Belgium and France, where the prices are freely negotiated, the discount ranges are 20–34% and 25–35%, respectively. Since 2014, the French Healthcare Products Pricing Committee (CEPS) has operated a dedicated pricing doctrine for biosimilars that ‘aimed at guaranteeing not only substantial savings but also the viability of use of such products’, as reported in its 2014/2015 annual report. In the French hospital setting, the doctrine is to decrease the reference medicine price by 10%, align the biosimilar price with the reference medicine price, and revise the biosimilar price in the subsequent price review (1 year later); this system was set up to avoid disadvantaging the biosimilar compared with its reference medicine invoiced on top of the diagnosis-related group (DRG) in the tendering process, as French hospitals are able to recover 50% of the difference between the price obtained after the call for tenders and the tariff set by the CEPS.

In the French outpatient setting, the CEPS does not define a reference price decrease, but states that the price decrease of biologic reference medicine should not be less than 15% (to reach 20% over time) and that the price decrease for biosimilars should be at least 30%, compared with the initial price of the reference medicine [28,39].

In Germany and the UK, the prices are set by manufacturers and the following discounts usually apply: 20–25% (depending on the class of the medicine) [40] and 10–25%, respectively. In Spain, biosimilar prices are generally discounted by 30%, although this is not mandatory [41]. In Sweden, only prices lower than those of the reference drugs are accepted.

#### Price re-evaluation [26]

In all countries, the prices of pharmaceuticals are periodically revised. Policies that could specifically impact the prices of biosimilars were found in Belgium, Germany, and Hungary. In Belgium, there is a mandatory price cut of 7.5%, applied to biologic drugs (including biosimilars) once the active ingredient has been reimbursed for 18 years [42]. In Germany and Hungary, reference prices applicable to biosimilars are periodically revised [43].

#### Tendering [19,23,26,44–48]

Tendering processes for biologics are in place in all surveyed countries, except for Greece where biosimilars are excluded from tenders, according to a specific circular [49]. Tenders are generally organized at hospital level in all countries of interest, except for Germany, where tendering operates mainly in the outpatient setting (managed by statutory sickness insurance funds). Outpatient tenders are also reported in Hungary, Poland, and the UK. These tenders cover either a whole therapeutic area or a group of drugs with the same active ingredient, or both. Award criteria are generally driven by the lowest price, but other criteria may also be taken into account in Belgium, France, Spain, and the UK, including additional services (e.g. medicine training or delivery service). In Hungary, a biologic tender (called biolicit, i.e. preferred biologic product) was introduced in the ambulatory setting in 2011, with blind bidding through an electronic system. Pharmaceuticals selected through the biolicit process (two brands may win) can be used for new patients with a 1 EUR co-payment; other medicines will have different levels of co-payment, depending on their price [50]. In Poland, outpatient (but also hospital) tenders are mandatory when the purchase value exceeds 30,000 EUR, and cover a group of drugs with the same active ingredient [51,52].

Of note, since the concerns regarding interchangeability still exist, non-exclusive tenders are widespread, except for in Poland, where exclusive tenders take place. Non-exclusive tenders result in inclusion of both the reference medicine and its biosimilar into the formulary, which allows physicians to decide about the medicine prescribed for a particular patient. As such, in Poland, where no special provisions regarding biosimilars apply, the results of tenders lead to treatment switches [53].

#### Other policies

In Belgium, a convention called ‘Future Pact’ was signed in July 2015 by the Minister of Social Affairs and Public Health, the pharmaceutical industry, and associations of physicians and pharmacists, aiming to encourage the use of biosimilars in at least 20% of new patients [54].

### Demand-side policies for biosimilars

An overview of key demand-side policies for biosimilars is presented in Table 4.

**Table 4.** Key demand-side policies for biosimilars.

Policy	Belgium	France	Germany	Greece	Hungary	Italy	Poland^a^	Spain	Sweden	UK
Pharmaceutical prescription budgets	×	×	✓	✓	×	✓	×	✓	✓	✓
Prescription quotas	✓	×	✓	–	✓	✓	×	✓	✓	×
Monitoring of prescriptions patterns	✓	×	✓	✓	–	✓	×	✓	✓	✓
Financial incentives or penalties aimed at physicians	✓	×	✓	✓	×	✓	×	✓	✓	✓
Prescription conditions/guidelines	✓	✓	✓	✓	✓	✓	×	×	✓	✓
Switching^b^	✓	✓	✓	–	✓	✓	✓	✓	✓	✓
INN prescribing	×	×	×	×	×	×	✓	×	×	×
Biosimilar substitution	×	✓^c^	✓^d^	×	×	×	–^e^	×	✓^d^	×
Financial incentives or penalties aimed at pharmacists	✓	✓	✓	✓	✓	✓	✓	✓	✓	✓
Patient co-payment	✓	✓	✓	✓	✓	✓	✓	✓	✓	✓
Information and education^f^	✓	✓	✓	–	✓	✓	✓	✓	✓	✓

x: Absence of policy; ✓: presence of policy.

^a ^ Polish regulations do not differentiate between small molecule generics and biosimilars.

^b ^ Generally allowed under physician’s supervision; In Germany and Sweden, authorities allow switching for biosimilars coming from the same manufacturer.

^c ^ France is the first EU country to explicitly authorize by law biosimilar automatic substitution under specified conditions (automatic substitution not yet implemented in practice).

^d ^ Only for specific groups of biosimilars, i.e. produced by the same manufacturer.

^e ^ No regulation; automatic substitution may occur.

^f ^ Limited educational material related to the overall information about biosimilars found and generally not specifically targeting physicians, pharmacists, or patients. Education programmes/educational publications dedicated to physicians were found for Germany and the UK, and one brochure for patients published by Institute for Patients’ Rights and Health Education was found for Poland.

#### Policies directed at physicians [6,19,23,26,36,48,55,56]

Pharmaceutical prescription budgets or prescription quotas, potentially impacting biosimilar prescription, are in place in eight countries (Belgium, Germany, Greece, Hungary, Italy, Spain, Sweden, and the UK); in half of these countries, there are policies specifically targeting biosimilars (Germany, Hungary, Italy, and Sweden). In Germany, considered one of the most successful EU MSs in creating a positive attitude towards biosimilars [6], prescription budgets and quotas for biosimilars are set in ambulatory care. Each year, the regional physician associations agree the quotas with their sickness funds; therefore, minimal prescription quotas differ across the whole country. In Hungary, prescription quotas and targets for biosimilars were introduced through an electronic system facilitating prescription of the cheapest medicines. In Italy, several regional provisions related to biosimilars regulate biologic prescription and expenditure targets. For example, (1) in the Campania and Umbria regions, the main objective is to achieve a biosimilar utilization rate at least equal to the number of naïve patients, whereas in the Veneto region, the dispensed packages of biosimilars must constitute at least 60% of all dispensed packages of G-CSF, EPO, or hGH; (2) in Sicily, Veneto, Campania, and Tuscany, drug-naïve patients must be treated with cheaper biosimilars, and when patients are switched, the lower cost medicine should be chosen; any deviations from these rules should be justified by the prescriber [57–60]. In Sweden, county councils set quotas and target budgets for hospitals, applicable to various therapeutic classes, including biosimilars. In Spain, although some publications report a lack of quotas for biosimilars [55,56], specific indicators aimed at improving biosimilar uptake appear to have been recently set up in Madrid by the Madrid Health Service [6,61]. In Belgium, physicians in ambulatory care are required to meet prescription targets for low-cost drugs – and biosimilars are considered low-cost drugs if their reimbursed price is cut by 15% relative to the reference medicine price [62].

Drug prescription budgets, although not specific to biosimilars, are implemented in Greece and the UK. In Greece, prescribing caps for physicians, based on prescriptions issued in the previous year, were introduced in 2014. In the UK, prescription budgets for general practitioners (GPs) are set by Clinical Commissioning Groups (CCGs), and prescribing targets exist for those GPs who participate in the voluntary Quality and Outcomes Framework scheme [63,64]. Of note, a gain-share agreement has been reported in the UK between the University Hospital Southampton National Health Service (NHS) Foundation Trust and the local CCGs (2015) for the introduction of an infliximab biosimilar to fund a switching programme in patients with inflammatory bowel disease (IBD) (i.e. to fund the additional staffing needed to implement and monitor a safe switch programme), to incentivize the use of lower cost biosimilars and incur savings [65].

Monitoring of drug prescriptions has been introduced in all countries which implemented quotas or prescription budgets (for Hungary, no specific information was found in the literature). Dedicated controls related to biosimilars were reported for Belgium, Germany, Italy, and Sweden.

Two different approaches to enforcing drug (and biosimilar) prescription patterns are observed among the surveyed EU MSs; these involve either financial penalties towards prescribers when quotas or targets are not respected (e.g. Germany, Italy [58,59], and Belgium) or potential financial rewards for physicians who meet their targets (e.g. Italy [6], Sweden, some Spanish regions, and the UK [63,64]). In the Italian Campania region, a decree issued in July 2016 specified that 50% of a decrease in expenditure from 2015 to 2016 on drugs with certain ATC codes [B03XA (EPO), A10AE (insulins), L03AA (G-CSF), L04AB (anti-TNF), and somatropin] could be used by the hospital. These savings can be used as an extra fund for innovative high-cost drugs monitored by the AIFA (45%) and for improving the structure of the hospital centre (5%). On the other hand, any growth in the expenditure should be paid for directly by the hospital [66].

Prescription conditions or guidelines that apply to biosimilars are established in nearly all EU MSs. Various guidelines on biosimilars have been published by national regulatory authorities, regional authorities, or HTA bodies [e.g. the Federal Joint Committee (G-BA) in Germany; French Drug Agency (ANSM); AIFA, and the regional governments of Tuscany, Veneto, Campania, Umbria, and Sicily in Italy [58,59,67]; as well as NICE; and some county councils and the Drug Therapeutic Committee in the Stockholm region, Sweden [68,69]] and various scientific associations (e.g. the Belgian IBD Research & Development Group [70], French National Society of Gastroenterology [71], Spanish Society of Rheumatology [72], and British Society for Rheumatology [73]). In most cases, these guidelines focus on the issues of switching and substitution, which are highly debated topics (switching and substitution are discussed in the next section), as well as on the conditions of biologic/biosimilar prescription, especially the target population (treatment-naïve vs previously treated patients), indications specified in the marketing authorization, any reimbursement restrictions, and/or recommendations based on cost and, in some cases, effectiveness.

In terms of the guideline landscape in individual countries, in Germany, biosimilars were included in clinical guidelines by physicians’ associations and the G-BA very early on. In Greece, pharmaceutical drug regulation obliges physicians to prescribe biosimilars strictly in accordance with indications and dosages approved in their marketing authorization. In Hungary, prescribers must initiate treatment of new patients with biologics that are up to 5% more expensive than the cheapest available one. In Poland, treatment-naïve patients with IBD should be treated with a biosimilar [74]. In Italy, several regions recommended that physicians should use biosimilars as a first line treatment for new patients; in the case of EPOs, a treatment plan (the Piano Terapeutico) must be completed [59,67,75]. In a position paper published in September 2016, AIFA stated that biosimilars have the same risk–benefit ratio as the reference medicine, and thus constitute a valid therapeutic option not only for treatment-naïve but also for experienced patients [76]. In Sweden, some county councils recommend replacing original biologics with the most cost-effective pharmaceutical when starting treatment [69]. Furthermore, in the Southern Sweden Healthcare Region, an agreement between three specialists was needed to initiate treatment with Neupogen® (the original filgrastim); now, as biosimilars are available, a single prescriber is permitted to start the therapy. In the UK, NICE has issued positive recommendations for biosimilars; for example, listing Omnitrope® (somatropin biosimilar) as one of the seven recommended somatropin medicines, and stating that where more than one somatropin medicine is suitable, the least costly option should be chosen (2010) [77–80].

Regarding switching between biologics, this is allowed under a physician’s supervision in most of the EU MSs. In France, the ANSM did not initially recommend switching of patients already treated with a biologic, to limit immunogenicity risk and ensure traceability for pharmacovigilance monitoring [81]. However, given the real-world evidence available for biosimilars, ANSM changed its position in May 2016, stating that switching could be considered during treatment if the patient is informed about the potential switch, his or her consent is obtained, and treatment is closely monitored [82].

In Belgium [70,83] and Spain [72,84], switching is not recommended in general, although it is allowed at the physician’s discretion. The Belgian Federal Agency for Medicines and Health Products (FAMHP) specified that no relevant changes in treatment were expected upon switching between a biologic reference medicines and its biosimilars, since biosimilars were approved based on having the same safety and efficacy profile as the reference medicine [83].

Hungarian guidelines permit switching between a suitable biosimilar and the original medicine in clinically justified cases (due to adverse events or a lack of efficacy), and at least 1 year after terminating the previous treatment [74,85]. In the UK, NICE left treatment choice to prescribers [30], but different – and often conflicting – recommendations are published by scientific associations; for instance, the British Society of Gastroenterology recommends switching those patients who are in a stable clinical response or remission on the reference medicine [86], while the National Rheumatoid Arthritis Society and the British Society for Rheumatology recommend that stable patients should not be switched [87]. In Italy, AIFA stated in its position paper that biosimilars were preferred if they constituted an economic advantage [88]. Poland is the only EU MS where switching is generally encouraged and can occur at every therapy level [89]; for example, since the launch of infliximab biosimilars, all patients in Poland are switched to biosimilars for the treatment of IBD [74]. In Germany and Sweden, switching is allowed for biosimilars coming from the same manufacturer. In Germany, switching is supported by the Paul-Ehrlich-Institut, as long as pharmacovigilance follow-up is ensured [90]. In Sweden, some county councils have started to recommend switching to biosimilars [69]. No rules related to switching were identified in Greece.

Finally, almost all surveyed EU MSs request brand prescribing (and not INN prescribing) for biologic medicines, except for Poland, where the choice is left to the prescriber and Polish law does not differentiate between generics and biosimilars.

#### Policies directed at pharmacists [19,23,26,36,91]

In the majority of investigated EU MSs, substitution of biologics by pharmacists is generally either not allowed or not recommended. France is the first EU country to explicitly authorize by law the automatic substitution of biosimilars under specified conditions (for medicines belonging to the same group, called the ‘similar biologic group’, at treatment initiation or to continue a treatment already initiated with the same biosimilar, when substitution is not prohibited by the physician, with information about substitution provided to the prescriber and the biosimilar name written on the prescription). However, an implementation decree is still pending and automatic substitution is currently not implemented in practice [92]. In other EU MSs, substitution is prohibited either by law (Belgium, Hungary, and Spain [93,94]) or by guidelines (Greece [95], Italy [88,96], and the UK [73,87]). In Germany and Sweden, substitution is generally not allowed but is possible for specific groups of biosimilars, i.e. those produced by the same manufacturer [97,98]. The situation is different in Poland, where automatic substitution may occur, as no specific regulations regarding biosimilars apply. In such circumstances, the Polish pharmaceutical association INFARMA listed on its website [99,100] several threats connected with treating biologic medicines in the same way as small molecules. In response, the Polish Ministry of Health published a statement that any substitution is acceptable during treatment with a biologic medicine [101].

All surveyed EU MSs have introduced several financial incentives for pharmacists to enhance dispensing of less expensive drugs; however, these do not appear to be specific to biosimilars. Such regulations have the potential to stimulate biosimilar penetration, but only when accompanied by automatic substitution. Regressive financing, which provides higher mark-ups for cheaper medicines, has been introduced in Belgium, France, Hungary, Italy, Poland, and Sweden. In addition, price-independent fees for generic dispensing exist in Belgium, France, Hungary, Germany, Sweden, and the UK. Claw-back systems or mandatory rebates are in place in Germany, Greece, Italy, Spain, and the UK [102–104].

#### Policies directed at patients [23,26]

While patient co-payments are in place in all surveyed countries, their level differs across the EU MSs. Specific co-payment systems favouring the use of cheaper medicines are established in five countries (Germany, Hungary, Poland, Spain, and Sweden). In Germany [43] and Poland, patients have to pay the difference between the retail price and the reference reimbursement price. In Sweden, if the original medicine is chosen instead of a biosimilar, patients have to cover the price difference between the reference medicine and the biosimilar. In Hungary, higher co-payments apply to ‘non-preferred’ medicines, and in Spain a 100% co-payment is charged for drugs whose price exceeds the reference one.

#### Information and education [6, 23,36]

Apart from the prescription guidelines discussed above, limited educational material broadly related to biosimilars was found; this generally did not specifically target physicians, pharmacists, or patients. Education programmes and educational publications dedicated to physicians were found in Germany and the UK, and one brochure for patients published by the Institute for Patients’ Rights and Health Education was found in Poland [105]. In Germany, the statutory health insurers and regional physicians’ associations support physicians in building trust in biosimilars, by conducting education campaigns, holding discussions, and publishing letters highlighting wider access to high-cost medicines and potential savings that they promise. In the UK in 2015, NHS England published a biosimilar guide, mainly to inform finance and procurement discussions regarding biosimilar medicines, but also to target physicians ‘to provide an update about the developing role of biosimilar medicines in the NHS in England and to support the safe, effective and consistent use of all biological medicines, including biosimilar medicines, to the benefit of patients’ [106]. Moreover, success stories about savings due to biosimilars have been published by UK hospitals [36]. Finally, the Adoption and Impact Programme publications from NICE, which aim to share experiences of introducing biosimilars within the NHS, may further support information provision and education of physicians through case studies [65].

Recently, several generic associations have published position papers and information on biosimilars (FeBelGen in Belgium [107], GEMME in France [108], Pro Generika in Germany [109], GE in Hungary [103], AssoGenerici in Italy [110], BGMA in the UK [111], AESEG in Spain [112], and FGL in Sweden [113]), and some have even created biosimilar branches (Pro Biosimilars in Germany [114], BBA in the UK [115], and BioSim in Spain [116]).

## Discussion

Supply- and demand-side policies applied to biosimilars differ between and even within the selected countries. Those measures, together with differences among therapeutic classes, may help to explain differences in biosimilar uptake reported across the EU MSs. Indeed, differences in biosimilar uptake have been shown between the different pharmacological classes; among the first three old biosimilar classes on the market (EPO, G-CSF, and hGH), uptake of somatropin biosimilar is generally lower, explained by the fact that somatropin is usually prescribed to children and used in the long term, whereas epoetin and filgrastim are used for short-term treatment [117]. A recent analysis of biosimilar uptake, performed by IMS Health in 2015, showed differences in uptake between different therapeutic classes and EU MSs. For example, among countries surveyed in this research, the highest market share of biosimilar EPO versus reference medicine (>90%) was reported in Greece, Poland, and Sweden, while the lowest was reported in Belgium (0%) and the UK (5%). In the case of G-CSFs, the highest biosimilars market share was observed in Hungary (100%), followed by Greece, the UK, and Sweden (>90%), and the lowest in Belgium (1%). When considering the market share of hGHs, Poland had the highest biosimilar share (99%), with a share of 32% or less in the remaining EU MSs. On the anti-TNF market, the highest share of biosimilars was also seen in Poland (78%), compared with 25% or less in the rest of the EU MSs. In Germany, which is known to have the most demand-side policies towards biosimilars of all EU MSs, the market share of biosimilar EPO, G-CSFs, hGHs, and anti-TNF versus reference medicines was 69%, 78%, 26%, and 10%, respectively [118].

Biosimilars should be distinguished from generics, especially in terms of higher development costs, owing to the complexity and high cost of the manufacturing process, and different development requirements [48,119]. Despite significant differences between biosimilar and generic markets, similar trends have been observed with regard to the factors potentially influencing generic and biosimilar uptake. The supply-side regulations targeting price (i.e. IRP, ERP, tendering, and mandatory price linkage) in general seem to limit the penetration of biosimilars in the long term, despite short-term savings. In contrast, demand-side policies are considered to impact uptake positively [119–124].

Supply-side policies – such as IRP, ERP, price linkage, and tendering for pricing and procurement of biosimilars – aim to push biologic reference medicine and biosimilar prices down to generate savings. However, significant compulsory price cuts on biosimilars are considered disincentivizing for manufacturers. To compete with biosimilars, reference medicine manufacturers would be tempted to cut their prices to maintain the uptake of branded biologics, thus reducing competition and decreasing the revenue profitability of biosimilar producers. Decreasing competition following biosimilar entry would ultimately lead to price increases in the longer term. One example of a way to avoid disadvantaging biosimilar competition is seen in France, where CEPS decided to align biosimilar price with reference medicine price in the hospital setting, and hospitals are able to recover 50% of the difference between the price obtained after the call for tenders and the tariff set by the CEPS. Tendering may also hamper biosimilar competition and discourage biosimilar producers, leading to a monopolistic market situation with a risk of shortages [36,55,125]. Finally, ERP, as already reported for generics, may limit patient access to biosimilars in some countries (especially where procurement and tendering systems are also in place) by driving down the prices to levels that may prevent launches by the biosimilar industry [27].

When HTA is performed for biosimilars, the assessment generally follows that of the reference medicine, if it is already reimbursed in the country. HTA may contribute to recognition of the value of biosimilars, especially in countries where HTA is driven by cost-effectiveness. In markets where the reference medicine is restricted for reimbursement to specific patient subgroups or not reimbursed at all, improvements in the cost-effectiveness ratios of biologics through biosimilar launches could alleviate the restrictions. For example, this was seen in the UK, where NICE recommended infliximab in the treatment of ankylosing spondylitis following infliximab biosimilar entry, while the reference medicine had not been initially recommended [79,126].

This study shows that, in nearly all EU MSs, the decision to change treatment has to be made under the responsibility of the physician; thus, the prescribers’ positive attitude and trust in biosimilar medicines should be the priority. Meanwhile, several studies have shown that physicians’ familiarity with biosimilars is not satisfactory and that prescribers perceive the information on biosimilars provided as not sufficient and even not trustworthy [6,127–131]. HTA may create the opportunity to satisfy the need for physicians’ education through guidance publication, which is shown to be crucial for the acceptance of biosimilars, where concerns regarding efficacy and safety may exist among healthcare professionals and patients [6,132].

Demand-side policies, i.e. physician, pharmacist, and patient incentives targeting biosimilars, are expected to increase uptake. As shown in a quantitative analysis [17], such policies remain an important driver for biosimilar penetration. Pharmaceutical prescription budgets and prescription quotas, combined with a monitoring system and financial incentives or penalties, have been reported as potential measures to stimulate biosimilar uptake [36,55,125]. For example, those policies have been proven to be effective in Germany, which has relatively high biosimilar uptake rates (up to 78% for G-CSFs) and has implemented several demand-side policies (e.g. prescription budgets, quotas, financial incentives, and information campaigns). Although changes in the rules or the position on switching and substitution of biosimilars have been made in some countries, biosimilar substitution is currently not allowed or not recommended in most countries, and the choice between treatment with a reference biologic or a biosimilar remains the responsibility of the prescribing physician. With no automatic substitution, pharmacist incentives are not expected to have a significant impact on biosimilar uptake. Recent studies among prescribers and payers have shown the necessity for improving the understanding of benefits connected with the use of biosimilars, and physicians’ trust in these drugs. Efforts are being made to enhance awareness of biosimilars, as more and more position statements related to biosimilar use are being published by scientific societies, together with the recommendations of HTA agencies in some EU MSs, especially aiming to overcome the concerns related to switching. Moreover, several ‘switching clinical studies’ have been carried out or are ongoing, with the objective of demonstrating the safety of switching [133–135]. The results of those studies, along with the requirements of pharmacovigilance applying to biosimilars, are likely to build confidence among physicians and patients, and stimulate demand. Switching is highly scrutinized by regulators and payers, and may be a lever for biosimilar uptake while automatic substitution is prohibited in most countries. Data regarding educational measures and information campaigns currently in place were generally scarce from the literature, so it is difficult to determine the efforts of national governments to enhance biosimilar adoption by different stakeholders.

## Conclusions

This study provides a comprehensive overview of biosimilar supply-side and demand-side policies implemented in selected EU MSs. As seen for generics, biosimilar policies vary widely among EU MSs, which may explain variations in uptake across the countries. Supply-side policies targeting price have been reported to limit the penetration of biosimilars in the long term, despite short-term savings, while demand-side policies are considered more effective at positively impacting uptake.

Biosimilars of biologic medicines, in contrast to generics of small molecules, require a higher financial investment for their development. Also, unlike for generics, the substitution of biologics by pharmacists is not recommended in the majority of the surveyed EU MSs. This contributes to the very specific biosimilar market dynamics. The biosimilar industry is still in the early stages of development, and manufacturers in this field need reassurance on the sustainability of their business model. Payers aiming at lowering biosimilar prices may increase manufacturers’ and investors’ concerns over investing in the biosimilar industry, slowing down the development of this market and preventing the achievement of the expected societal and health insurance benefits related to biosimilar uptake.

The biosimilar market is expected to contribute to the improved efficiency of healthcare systems by expanding access to affordable biologics and contributing to the financial sustainability of health insurance budgets. These expectations may be reached if health policies encourage biosimilar uptake and improve industry attractiveness, while increasing savings.

Understanding that a return on investment is needed to ensure the sustainability of the biosimilar industry is critical for achieving both the economic savings associated with biosimilar entries and the health benefits associated with a wider use of biologics.

Further research is warranted to inform biosimilar supply- and demand-side policies within the EU. A sustainable biosimilar industry will allow fulfilment of the societal value promises for these medicines.
